# Reply to Trexler, E.T. Common Errors in Sports Nutrition Meta-Analyses Lead to Distortion of Pooled Effect Estimates. Comment on “Viribay et al. Effects of Arginine Supplementation on Athletic Performance Based on Energy Metabolism: A Systematic Review and Meta-Analysis. *Nutrients* 2020, *12*, 1300”

**DOI:** 10.3390/nu17142376

**Published:** 2025-07-21

**Authors:** Aitor Viribay, Julen Fernández-Landa, Jesús Seco-Calvo, Juan Mielgo-Ayuso

**Affiliations:** 1 Glut4Science, Physiology, Nutrition and Sport, 01004 Vitoria-Gasteiz, Spain; aitor@glut4science.com; 2 Department of Physical Activity and Sports, Faculty of Education and Sport, University of Deusto, 48007 Bilbao, Spain; julenfdl@hotmail.com; 3 Department of Nursing and Physiotherapy, University of Leon, 24071 Leon, Spain; dr.seco.jesus@gmail.com; 4 Institute of Biomedicine (IBIOMED), Physiotherapy Department, University of Leon, Researcher of Basque Country University, Campus de Vegazana, 24071 Leon, Spain; 5 Department of Health Sciences, Faculty of Health Sciences, University of Burgos, 09001 Burgos, Spain

We deeply appreciate the time and effort invested by the comment author [1] in reviewing our meta-analysis on arginine supplementation and its effect on athletic performance [2]. We recognize that the points raised are relevant and that the observations about common errors in meta-analyses within the field of sports nutrition are very accurate.

Indeed, as noted in the commentary, the presence of outliers and confounding between standard errors and standard deviations are problems that can inflate effect size estimates. We appreciate the identification of these errors and would like to confirm that, in the subsequent analyses we have performed, we have implemented more rigorous procedures to deal with these outliers. In these new meta-analyses, we have corrected the distinction between standard errors and standard deviations, ensuring the inclusion only of data that do not distort the final result.

Regarding the use of standardization of mean differences, we have carefully reviewed the application of standardization techniques for both raw and change scores in our future studies. In our recent work, we have opted to use a single type of standardization across all included studies, which improves the consistency of the metrics and allows for clearer interpretation of the results.

Finally, regarding the handling of within-study correlations, we have also adopted a more rigorous approach to adjusting dependent effects between within-study measurements, as recommended in the current literature. This adjustment has been implemented using multivariable modeling methods and aggregating effect sizes where necessary.

We would like to point out that the aforementioned problems, such as poorly managed outliers, inconsistencies in the standardization of mean differences, and lack of adjustment of within-study correlations, are not unique to our analysis, but are common errors that occur in many meta-analyses in the field of sports nutrition, as documented in the work of Kadlec et al. (2023) [3]. In this regard, our more recent works already address these issues in a more rigorous manner. For example, in the articles by Fernandez-Landa et al. (2024) [4] and (2023) [5] and Santibañez-Gutierrez et al. (2023) [6], these methodological problems have been corrected and a more robust approach to data handling and calculation of effect sizes has been implemented.

We believe that an article that more generally addresses these errors rather than focusing solely on a single meta-analysis would be very valuable, as it would help to improve the quality of research in the area and would be beneficial to the scientific community in general.

We reiterate our appreciation for the constructive comments. The suggestions provided have been instrumental in improving the quality of our subsequent meta-analyses and ensuring that the results are more robust and useful to the scientific community. We will continue to apply these best practices to improve the accuracy and validity of our future work.
